# Striking a balance: outcomes of short-term Mono-J placement following ureterorenoscopy

**DOI:** 10.1007/s00240-021-01264-4

**Published:** 2021-04-13

**Authors:** Alina Reicherz, Verena Maas, Moritz Reike, Mirco Brehmer, Joachim Noldus, Peter Bach

**Affiliations:** 1 Department of Urology, Marien Hospital, Ruhr-University Bochum, Hölkeskampring 40, 44625 Herne, Germany; 2 Department of Urology, Augusta Medical Center Bochum, University of Witten/Herdecke, Bochum, Germany

**Keywords:** Ureteroscopy, Urolithiasis, Transient ureteral stenting using an external ureteral catheter, Mono-J

## Abstract

To evaluate factors affecting the outcomes of short-term Mono-J insertion for 6 h following ureteroscopic stone removal. Patients treated with a Mono-J for 6 h after ureterorenoscopy and stone removal were analysed. FaST 1 and 2 (Fast Track Stent Studies), two consecutive single academic centre studies, were conducted between August 2014 and April 2018. In each study, we randomized patients with renal or ureteral calculi to two groups before ureterorenoscopy. FaST 1 compared a Mono-J insertion for 6 h versus Double-J insertion for 3–5 days after ureterorenoscopy. FaST 2 compared a Mono-J insertion to a tubeless procedure in the same clinical setting. All patients were pre-stented for 3–5 days before URS. The study endpoint was stent-related symptoms as assessed by a validated questionnaire (USSQ). Results were stratified by clinical parameters, stone characteristics and operation details. 108 of 156 initially randomized patients undergoing ureterorenoscopy were included. USSQ scores covering the time 3–5 weeks after stone removal showed a significantly reduced urinary symptoms and pain index compared to the scores before ureterorenoscopy. USSQ results before and after stone removal did not correlate with stone size or operation time and did not differ significantly depending on stone localization, the treating endourologist, or ureterorenoscopic device used (*p *> 0.05). Six patients (5%) required reintervention. Following secondary ureterorenoscopy and ureteral drainage with a Mono-J for 6 h, quality of life is independent of stone size and localization, operation time, the treating endourologist, and the URS device used.

**Supplementary Information:**

The online version contains supplementary material available at 10.1007/s00240-021-01264-4.

## Introduction

The first ureterorenoscopy (URS) was performed about 40 years ago. In the middle of the 1990s, urologists began questioning the need for post-URS Double-J (DJ) stenting. It remains a matter of debate to the present day, as the majority of patients complain about stent-associated pain and severe micturition problems. A Cochrane analysis published in 2019 on post-URS stenting revealed that most studies on the topic are limited by retrospective design and small sample size, limiting the ability to determine best practices [1]. Insertion of stents ensures urine drainage and promotes the healing of ureteral lesions after URS; however, they are known to cause stent-related symptoms leading to higher postoperative morbidity and increased costs [2–4].

European and American guidelines do not recommend routine DJ insertion after uncomplicated URS [5, 6]; nevertheless, many urologists frequently use stenting. Our research group’s empirical study showed that after primary URS, German urological departments insert a DJ in 79.6% of cases, a Mono-J (MJ) in 7.3% and only 3.6% prefer tubeless procedure. After secondary URS, departments insert a DJ in 62.2% of procedures, a MJ in 10.5% and omit stenting in 14.0% [7]. Mittakanti published an observational study including 17,129 patients from North America in 2018. Patients undergoing laser lithotripsy received a DJ in 86.2% of cases and those undergoing basket retrieval in 70.5% [8].

The Cochrane analysis showed that secondary interventions were necessary for 3 out of 1000 patients when a DJ was inserted after URS, and in 21 out of 1000 when stenting was not performed.

Do urologists encounter problems when omitting to stent more often than the literature indicates? Khanna et al. reported that of 151,006 patients who underwent URS, 10.6% visited the emergency department postoperatively [9].

Striking a balance would be favourable, as it is difficult to predict which patients will require stenting. Based on Moon et al.’s editorial comment, the EAU guideline states that surgeons can insert a ureteral catheter for 1 day with similar results to long-term stenting [6, 10]. To our knowledge, besides the prospective randomized FaST studies from our research group, the retrospective study by Merlo et al. is the only approach to evaluate a short-term external MJ after URS [11].

The FaST 1 and 2 studies comprised 108 pre-stented patients who underwent MJ insertion following URS [12, 13]. Patients were prospectively randomized into two groups: DJ insertion for 3–5 days versus MJ insertion for 6 h in FaST 1, and MJ insertion for 6 h versus tubeless procedure in FaST 2. We were able to demonstrate that patients receiving an MJ and patients who do not receive a tube benefit from a significantly improved QoL, compared to those undergoing routine DJ insertion, through the validated tool (USSQ), however, at the cost of an increased reintervention rate when stenting is omitted.

Ultimately, the need to stent relies on the urologists’ assessment. A MJ placement fixes the surgeon’s dilemma of no drainage when stenting is omitted and DJ-stent-associated morbidities.

The present work aims to ease the decision regarding stenting by analysing which factors influence outcomes after URS procedure and a short-term MJ insertion.

### Patients and methods

Patients from the FaST studies who received a MJ for 6 h after URS and stone removal were included. The single-center FaST 1 and 2 studies recruited patients from August, 2014 to April, 2018. The Ruhr-University of Bochum obtained ethics approval.

Fast 1 assigned patients via block randomization to DJ insertion for 3–5 days or short-term MJ insertion (6 h postoperatively); FaST 2 randomized to short-term MJ drainage (treatment analogous to FaST 1) or tubeless procedure after URS procedure. The allocation ratio was 1:1. Inclusion criteria were age at least 18 years and ureteral or renal stones smaller than 25 mm. Exclusion criteria were a single kidney or concurrent urinary tract infections (UTI). Secondary exclusion criteria were AAST Grade 2 complications (American Association for the Surgery of Trauma-Organ Injury Scale) including ureteral extravasation, an operation time of more than 60 min and a stone-free rate less than 80% after initial stone removal. At anaesthesia induction, all patients got a single dose of prophylactic antibiotics. Thirteen surgeons, not blinded to the randomization, participated in URS procedures. Surgeons used either a 6.4 Fr and/or 4.2 Fr channel semirigid URS instrument (Olympus^®^) and a 9.9 Fr flexible URS instrument (Olympus^®^) at their discretion. If necessary, surgeons used a LisaLaser^®^ for laser lithotripsy and basketing technique for stone retrieval. Treating urologists assessed the stone-free rate (SFR) after the procedure by X-ray. A VORTEK^®^ 6 Fr, Single loop ureteral stent (Coloplast^®^) was used for short-term ureteral stenting. The MJ was placed via a foley with a whistle tip to prevent it from dislocating [14]. The MJ and foley were removed after 6 h on the ward by a nurse without anaesthesia.

Standard analgesic treatment comprised 50 mg diclofenac orally twice daily and Tamsulosin 0.4 mg (off label) orally once daily for 3 days after URS.

The primary outcome measures were stent-related symptoms assessed by the validated German Ureteral Stent Symptom Questionnaire (USSQ), which was self-administered by patients both prior to (I) and 3–5 weeks (II) after URS [15]. The secondary endpoint was the reintervention rate. To demonstrate a difference of 15% in the urinary symptoms index between populations with a statistical power of 85%, a sample size of 60 patients in each group was required^9^. We applied an intention-to-treat analysis and deleted listwise if data were missing. We used the software GraphPad Prism 5 for statistical analysis. We compared USSQ results using a Mann–Whitney *U* test and assessed the correlation between USSQ results and collected parameters using Spearman correlation. For multivariate analysis of parameters and USSQ results, we utilized the Kruskal–Wallis test. Fisher’s exact test was used to evaluate reintervention rates. The level of significance was defined as *p* < 0.05.

## Results

**Fig. 1:**
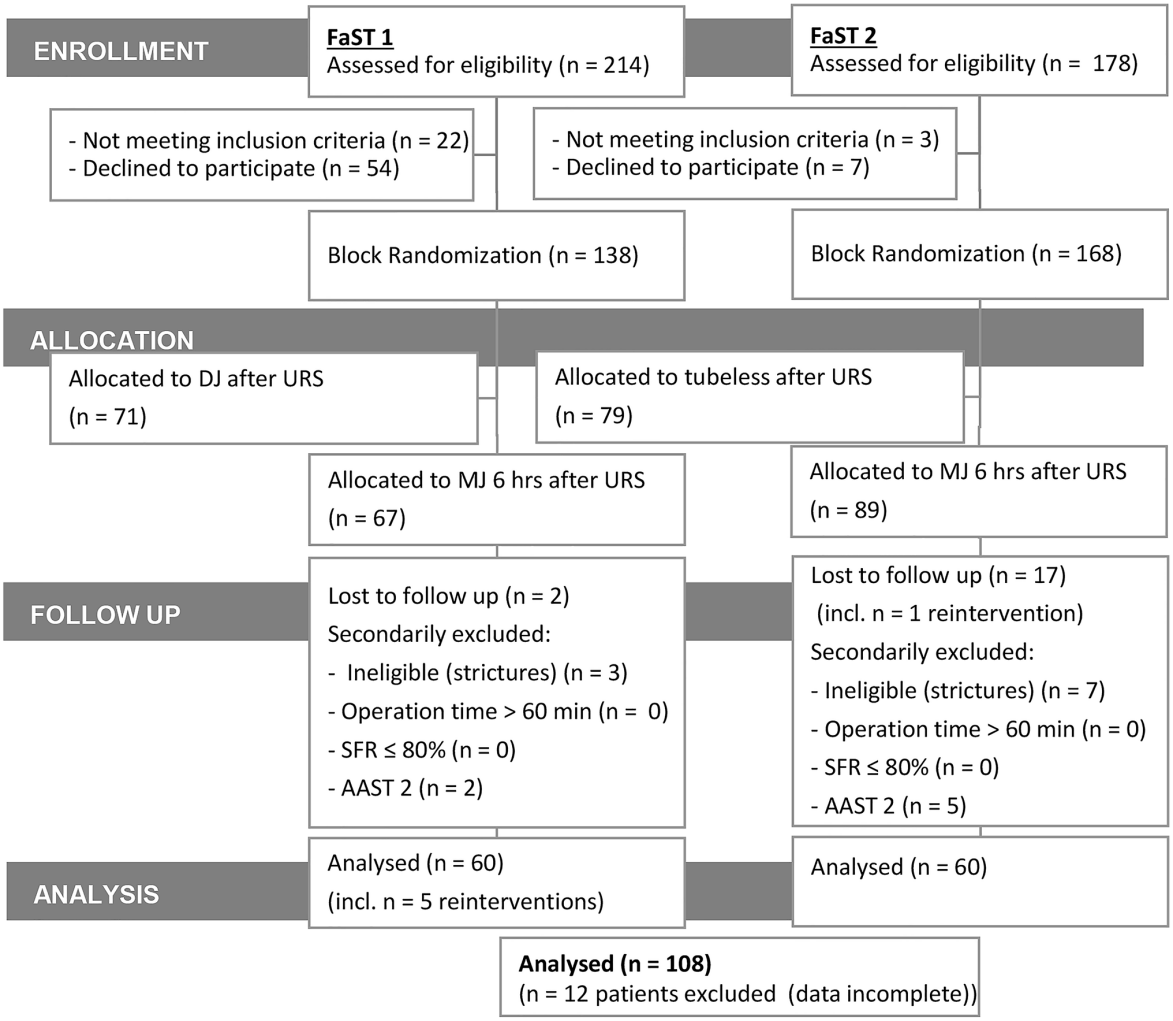
CONSORT diagram: study flow diagram of the progress through the phases in the FaST 1 and 2 studies

FaST 1 assessed 214 patients for eligibility, of which 138 were block-randomized, and 67 were allocated to MJ insertion for 6 h after URS (Fig. 1). FaST 2 assessed 178 patients for eligibility: 168 were block-randomized, and 89 were allocated to MJ insertion for 6 h after URS. 19 patients were lost to follow-up. In FaST 1, 5 patients were secondarily excluded due to ineligibility because of strictures or AAST 2 complications. In FaST 2, we secondarily excluded 12 patients due to ineligibility or AAST 2 complications. Five patients required reintervention in the FaST 1 group and one patient in the FaST 2 population: reasons were therapy-resistant pain caused by symptomatic hydronephrosis or infection (> 38.5 °C). In these cases, a DJ was inserted. 21 patients were lost to follow-up. In 12 patients, USSQ results were missing and deleted listwise. In summary, we analysed 108 patients who had received a MJ for 6 h after URS and stone removal. Table 1 shows the patients' characteristics.

**Table 1:** Patients’ characteristics

	Mean (± SEM)
Gender (%)	
Male	74.1
Female	25.9
Age (years)	48.7 ± 1.4
BMI (kg/m^2^)	28.5 ± 5.0
Stone size (mm)	6.1 ± 3.9
Stone location (%)	
Distally	27.8
Lumbar	24.1
Proximally	25.0
Nephrolithiasis	7.4
No stone found	15.7
Operation time (min)	21.1 ± 12.3
Flexible URS (%)	42.6

### USSQ scores

1 day before URS, patients reported their urinary symptoms using the USSQ (Table 2). These complaints, displayed in the urinary index, significantly reduced 3–5 weeks after stone removal (*p* < 0.0001). 1 day before URS, 84.3% of patients described pain caused by the indwelling DJ. The incidence of pain was reduced to 25.0% 3–5 weeks after stone removal, as well as the intensity of pain; the pain index reduced significantly (*p* < 0.0001). The general health index showed that patients had few difficulties in their everyday performance 3–5 weeks after stone treatment (Table 2). After URS procedure and short-term MJ insertion, patients were rarely immobilized (on average 0.3 days) or had to cut down their usual activities (0.8 days). If patients were asked how they would feel in the future if they were advised to have another stent inserted, they answered to feel "mostly satisfied".

**Table 2:** USSQ results of FaST 1 and 2 patients 1 day before and 3-5 weeks after URS and MJ insertion for 6 hours

	Before URS [1]	After URS [2]
	Mean + SEM	
Urinary symptoms index	29.5 ± 7.7	18.1 ± 6.2
Pain index	23.2 ± 11.3	9.4 ± 8.1
General health index	–	9.2 ± 4.3
GQ^†^	–	2.7 ± 1.5

[1] Questioning 1 day before URS

[2] Questioning 3–5 weeks after URS and MJ insertion for 6 h

^†^Question GQ: “In the future, if you were advised to have another stent inserted, how would you feel about it?”

### Influence of patient characteristics on USSQ results

The mean body mass index (BMI) was 28.5 kg/m^2^ ± 5.0. No correlation was observed between BMI and the urinary symptoms index at the first questioning (1 day before URS 95% CI − 0.12 to 0.27) or the second questioning (3–5 weeks after URS 95% CI − 0.12 to 0.26). A correlation between BMI and the pain index was not detected either during the first (95% CI − 0.27 to 0.12) or the second questioning (Table 3).

**Table 3:** Influence of patient and stone characteristics and operation  parameters on USSQ results before and after URS

	p Value/95% CI					
	Before URS [1]		After URS [1]			
	Urinary index	Pain index	Urinary index	Pain index	General health index	GQ^†^
Sex	0.66	0.64	0.77	0.41	0.39	0.37
BMI	− 0.12–0.27	− 0.27–0.12	− 0.12–0.26	− 0.09–0.30	− 0.06–0.32	− 0.08–0.30
Recurrent stone disease	0.30	**0.04**	0.75	0.75	0.52	0.50
Stone size	− 0.16–0.26	0.34–0.08	0.20–0.22	− 0.09–0.33	− 0.11–0.31	− 0.23–0.18
Stone localization	0.82	0.14	0.97	0.33	0.54	0.72
Operation time	–	–	− 0.15–0.24	− 0.12–0.27	− 0.13–0.26	− 0.30–0.06
Surgeon	–	–	0.99	0.33	0.51	0.52
URS device	–	–	0.27	0.50	0.12	0.76

Mann–Whitney *U* test for comparison between USSQ results and sex/recurrent stone disease/surgeon/URS device; Pearson correlation of results and BMI/stone size/operation time; Kruskal–Wallis test for USSQ results and stone localization

Bold indicates significant value is 0.04

[1] Questioning 1 day before URS

[2] Questioning 3–5 weeks after URS and UC insertion for 6 h

^†^Question GQ: “In the future, if you were advised to have another stent inserted, how would you feel about it?”

25.9% of the patients in the MJ arms were female, respectively, 74.1% were male. Patients experienced urinary symptoms, pain, and general health problems independent of their sex (Table 3). No significant difference was observed between males and females regarding the urinary symptoms index at the first questioning (*p* = 0.65) and the second questioning (*p* = 0.77). The pain index did not differ significantly throughout the treatment [(I) *p* = 0.64, (II) *p* = 0.41].

Independently of whether patients had suffered from recurrent stone disease, they experienced urinary symptoms [(I) *p* = 0.30, (II) *p* = 0.75]. However, patients who had a recurrent stone episode had a significantly higher pain index during pre-stenting (*p* = 0.04). 3–5 weeks after URS, this difference was no longer present [(II) *p *= 0.75]. Performance in daily life prompted in the USSQs general health domain did not differ regardless if it was the patient's first presentation or a recurrent stone [(II) *p *= 0.52]. Furthermore, the comparison of future expectations (question GQ) did not differ significantly between the groups [(II) *p *= 0.50] (Table 3).

### Influence of stones’ characteristics on USSQ results

Stones were located distally in 27.8% (30/108), lumbar in 24.1% (26/108), proximally in 25.0% (27/108) and in the pelvicalyceal system in 7.4% (8/108). In 15.7% (17/108) of cases, no stone was found, most likely due to a spontaneous stone passage. Multivariate analysis showed no significant difference in the urinary symptoms index depending on the stone's location (*p *= 0.97). Moreover, neither a relationship between stone localization and the pain index (*p *= 0.33) nor between stone localization and the general health index (*p *= 0.54) was detected (Table 3).

In the MJ population, stone size did not affect urinary symptoms, pain or general health complaints (Table 3). The mean stone size was 6.1 mm ± 3.9. The urinary index was not correlated with stone size either at the first (95% CI − 0.16 to 0.26) or second questioning (95% CI − 0.20 to 0.22). Also, the pain index and stone size did not correlate at both times surveyed [(I) 95% CI − 0.34 to 0.08; (II) 95% CI − 0.09 to 0.33].

### Influence of operation parameters on USSQ results

In our study population, no correlation was detected between the operation time and USSQ indices (Table 3). Mean operation time was 21.1 min ± 12.3. The length of time did not correlate with the urinary symptoms index after URS (95% CI − 0.15 to 0.24), the pain index (95% CI − 0.12 to 0.27) or the general health index (95% CI − 0.13 to 0.26).

URS were performed by 13 surgeons. USSQ results from patients who had been operated by the senior physician most experienced in stone therapy were compared to those of the rest of the surgeons. Patients described the same severity of urinary symptoms (*p *= 0.99) and pain (*p *= 0.33) regardless of who had performed the surgery (Table 3).

57.4% (62/108) of patients required rigid URS only. In 42.6% (46/108) of patients, endourologists utilized both a flexible and a rigid URS device for stone removal. The surgeons inserted an access sheath (12 Fr, Coloplast^©^) for flexible URS procedures. 3–5 weeks after stone removal, the urinary index (*p *= 0.29), pain index (*p *= 0.50) and general health index (*p *= 0.51) did not differ significantly according to the URS instrument used.

## Discussion

American, European and German guidelines state that routine stenting after URS and stone removal is not necessary. Despite the downsides of stenting, data from America and Germany indicate that urologists are reluctant to omit stenting [7, 8]. This hesitancy might be based on a higher risk of readmission. A systematic review and meta-analysis by Pais et al. reported that “the odds of hospital readmission were almost 4 times as high in patients without a stent vs patients with a stent (OR 3.75, 95% CI 2.09–6.74)” [16].

The prediction of which patients will require stenting after URS is challenging. The European guidelines on urolithiasis state that a ureteral catheter insertion for 1 day after URS and stone removal can be performed with similar results [6]. However, a clear basis for this recommendation can not be identified in the comment [10].

FaST 1 and 2 trials from our research group were the first prospective randomized trials comparing a short-term MJ insertion to either a DJ placement or tubeless procedure, respectively [12, 13]. Short-term MJ insertion after URS ensures immediate drainage and is not accompanied by lower stent-related symptoms due to a shorter indwelling time: FaST 1 and 2 trials showed that a short-term MJ insertion is safe (reintervention rate of 5.0%) and significantly reduces stent-associated symptoms compared to regular DJ stenting. An alternative to MJ insertion for short-term stenting is using a DJ with a tether or with a magnet attached to the distal coil. However, DJs with tethers are known to have an increased risk of dislocation [17]. To reduce the risk of dislocation, we placed the MJ via a foley with a whistle tip. None of the MJs in patients included in the study dislocated.

A DJ with a retrieval device (magnet) is commercially available at Urotech^®^. Sevcenco et al. prospectively investigated the morbidity, complication rate and pain perception in 151 patients associated with removing a DJ with a magnet [18]. They reported that stent removal was significantly less painful in patients with a magnet stent than in those with a regular stent (*p *< 0.001). However, stent irritation was slightly lower in those with a regular stent (*p *< 0.001). O’Kelly et al. compared 50 patients with a magnetic stent to 50 patients who were retrospectively asked to complete the USSQ regarding the removal of their DJ via flexible cystoscopy [19]. Pain caused by stent removal was significantly lower in those patients who had a DJ with a magnet. No significant difference was found regarding USSQ results or complication rates.

The presented analysis demonstrates that patients benefit from short-term stenting regardless of the clinical parameters, stones' characteristics and operations’ details.

Neither stone size nor location correlated with USSQ outcomes. However, it has to be considered that in line with the trial design, stones larger than 25 mm were excluded, and the mean stone size was 6.1 mm ± 3.9.

Moreover, the operation time did not correlate with USSQ results. An operation time of more than 60 min was an exclusion criterion.

The surgeon’s experience had no impact on the outcome in terms of USSQ results. Data from the BUSTER trials by Wolff et al. and retrospective data by Netsch et al. showed no difference in the stone-free and complication rates between residents and young specialists, nor between residents and senior physicians [20, 21]. However, Wolff et al. stated that experienced specialists' stone-free rates were significantly higher. Remarkably, specialists are more likely to insert a stent after URS than residents (69.7% versus 86.6%; *p *= 0.002) [20].

A valid comparison between the surgeon's experience and reintervention rates can not be drawn from the present analysis due to the low numbers of reintervention.

The URS device and usage of an access sheath had no significant impact on USSQ results. A prospective observational study from Italy showed the same effect after URS, stone removal and DJ insertion for 4 weeks [22]. A subgroup analysis between semirigid and flexible URS devices found no significant difference in answers to USSQ questions of the urinary, pain or general health domain 3 weeks after stone removal.

### Limitations

A potential source of bias is that surgeons were aware of randomization and that only patients stented before URS were randomized and included.

## Conclusion

We conclude that a MJ insertion with an indwelling time of 6 h is a safe procedure after secondary URS. Patients benefit from a significantly increased QoL compared to a DJ placement and from a short-term insertion by being tubeless on the evening of the surgery. All these benefits are regardless of stone size, location, the surgeon’s experience, operation time or the URS device used. Hence MJ insertion after secondary URS is the new standard in our department.

Further prospective studies are needed to evaluate the role of no and a short-term stent insertion before URS and stone extraction as well as the optimal time for short-term stenting after URS.

## Supplementary Information

Below is the link to the electronic supplementary material.

Supplementary file1 (PDF 91 KB)

Supplementary file2 (PDF 101 KB)

## Abbreviations

AAST: American association for the surgery of trauma
BMI: Body mass index
DJ: Double-J stent
FaST: Fast Track Stent Studies
MJ: Mono-J stent
QoL: Quality of life
SFR: Stone-free rate
UC: Ureteral catheter
URS: Ureteroscopy retrograde surgery
USSQ: Ureteral stent symptom questionnaire
UTI: Urinary tract infections
