# Supplementary material for: Striking a balance: outcomes of short-term Mono-J placement following ureterorenoscopy
Source: Urolithiasis. 2021 Apr 13;49(6):567–73. doi: 10.1007/s00240-021-01264-4 (PMC8560726; doi:10.1007/s00240-021-01264-4)
Supplement: Supplementary file 1 — Supplementary file1 (PDF 91 KB) [file 240_2021_1264_MOESM1_ESM.pdf]

URETERIC

STENT SYMPTOMS QUESTIONNAIRE

Questionnaire 2 (*Post stent*)

We are interested to know about various aspects of <u>your health following removal of the stent</u> and the impact removal of stent has had on your health.

Please complete the following questionnaire, which has different sections. Please answer all questions in each section.

(We would be grateful if you complete and post the questionnaire within seven days)

Please complete,

Today's Date: ☐☐ / ☐☐ / ☐☐

Date of Birth: ☐☐ / ☐☐ / ☐☐

***Please return to*:**

Post Code: ☐☐☐☐ ☐☐☐
Hospital Number: ☐☐☐☐☐☐☐☐
(for office use)

You will see that some questions ask if you have a symptom occasionally, sometimes or most of the time.

**Occasionally**	=	**less than one third of the time**
**Sometimes**	=	**between one and two thirds of the time**
**Most of the time**	=	**more than two thirds of the time**

URINARY SYMPTOMS

Please answer the questions thinking about the urinary symptoms you have experienced following removal of the stent.

Please put a tick in one box for each question ☑

Please think about your experience since removal of the stent.

U1. During the day, how often do you pass urine, on average?

- Less than hourly ☐ 5
- Hourly ☐ 4
- Every 2 hourly ☐ 3
- Every 3 hourly ☐ 2
- Every 4 hours or more ☐ 1

U2. During the night, how many times do you have to get up to pass urine, on average?

- None ☐ 1
- 1 ☐ 2
- 2 ☐ 3
- 3 ☐ 4
- 4 or more ☐ 5

U3. Do you have to rush to the toilet to urinate?

- Never ☐ 1
- Occasionally (less than one third of the time) ☐ 2
- Sometimes (between one and two thirds of the time) ☐ 3
- Most of the time (more than two thirds for the time) ☐ 4
- All of the time ☐ 5

U4. Does urine leak before you can get to the toilet?

- Never ☐ 1
- Occasionally ☐ 2
- Sometimes ☐ 3
- Most of the time ☐ 4
- All of the time ☐ 5

U5. Do you leak urine without feeling the need to go to the toilet?

- Never ☐ 1
- Occasionally ☐ 2
- Sometimes ☐ 3
- Most of the time ☐ 4
- All of the time ☐ 5

U6. How often do you feel that your bladder has not emptied properly after you have passed urine?

- Never ☐ 1
- Occasionally (less than one third of the time) ☐ 2
- Sometimes (between one and two thirds of the time) ☐ 3
- Most of the time (more than two thirds of the time) ☐ 4
- All of the time ☐ 5

U7. Do you have a burning feeling when you pass urine?

- Never ☐ 1
- Occasionally ☐ 2
- Sometimes ☐ 3
- Most of the time ☐ 4
- All of the time ☐ 5

U8. How often do you see blood in your urine?

- Never ☐ 1
- Occasionally ☐ 2
- Sometimes ☐ 3
- Most of the time ☐ 4
- All of the time ☐ 5

U9. How much blood do you see in your urine?

- Do not see any blood ☐ 1
- Urine is slightly blood stained ☐ 2
- Urine is heavily blood stained ☐ 4
- Urine is heavily blood stained and has clot(s) ☐ 5

U10. Overall, how much of a problem are your urinary symptoms to you?

- Not at all ☐ 1
- A little bit ☐ 1.25
- Moderate ☐ 1.5
- Quite a bit ☐ 1.75
- Extreme ☐ 2

U11 If you were to spend the rest of your life with the urinary symptoms, if any, associated with the kidney problem just the way they are, how would you feel about it?

- delighted ☐ 1
- pleased ☐ 2
- Mostly satisfied ☐ 3
- Mostly dissatisfied ☐ 5
- Unhappy ☐ 6
- Terrible ☐ 7
- Mixed feelings (about equally satisfied and dissatisfied) ☐ 4

Please go to next section --

BODY PAIN (for women):

This section asks about the **body pain or discomfort, which you associate with the kidney problem.**

Please think about your experience **following removal of the stent.**

P1. Do you experience body pain or discomfort in relation to your kidney problem?

YES ☐1, **please go to question P2**

NO ☐2, **please go to next section on General Health** (Ignore questions P2 to P9)

P2. Think of the drawings below as the drawings of your body. Please **mark (X) or shade the site(s) where you experience pain or discomfort in relation to your kidney problem typically (e.g. during the day to day activities, whenever you pass urine)**

If you get pain at more than one site, please use a separate mark for each site.

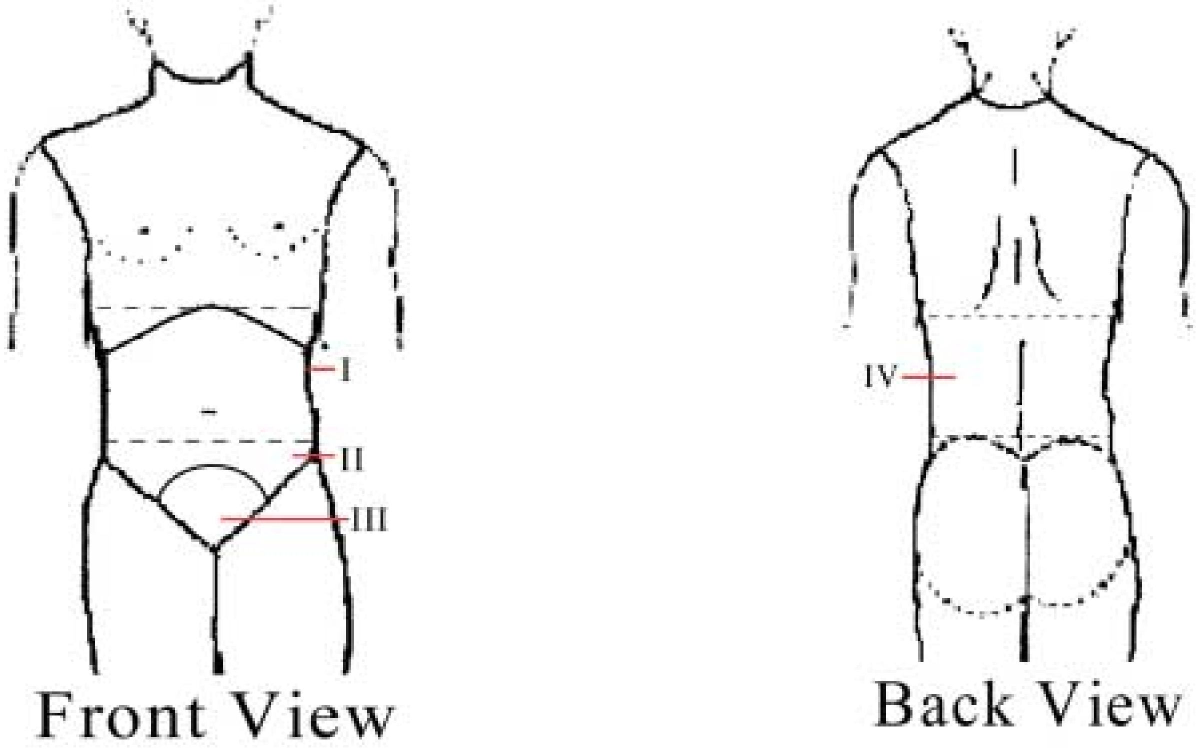

The numbers I - IV represent following areas for the right and left sides

I – Kidney front/side area
III – Bladder area
II - Groin area
IV– Kidney back (loin) area

Please use O for any other marked area and write the name of the site

P3. Please place a mark (X) to a point on the line below that indicates pain or discomfort in relation to your kidney problem. **Please put a separate mark for each site** if the pain or discomfort is different in severity and write the corresponding number of each site used in the drawing above.

0 1 2 3 4 5 6 7 8 9

No Pain or discomfort **Worst Possible Pain**

BODY PAIN (for men):

This section asks about the **body pain or discomfort, which you associate with your kidney problem.**

Please think about your experience **following removal of the stent.**

P1. Do you experience body pain or discomfort in relation to your kidney problem?

YES ☐1, **please go to question P2**

NO ☐2, **please go to next section on General Health** (Ignore questions P2 to P9)

P2. Think of the drawings below as the drawings of your body. Please **mark (X) or shade the site(s) where you experience pain or discomfort in relation to your kidney problem typically (e.g. during day to day activities, whenever you pass urine)**

If you get pain at more than one site, please use a separate mark for each site.

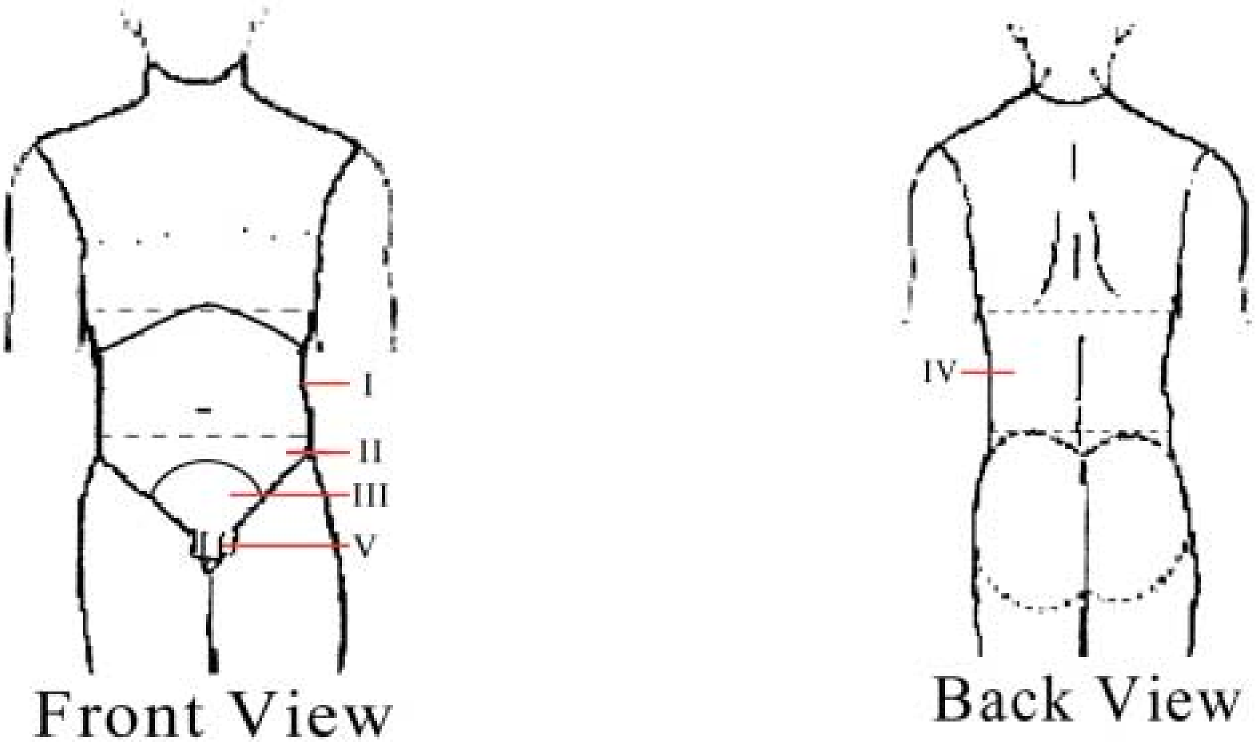

The numbers I - V represent following areas for the right and left sides

I – Kidney front/side area
II - Groin area
III – Bladder area
IV– Kidney back (loin) area
V - Penis

Please use O for any other marked area and write the name of the site

P3. Please place a mark (X) to a point on the line below that indicates your pain or discomfort in relation to your kidney problem. **Please put a separate mark for each site** of the pain or discomfort is different in severity and write the corresponding number of each site used in the drawing above**.**

0 1 2 3 4 5 6 7 8 9 10

No Pain or discomfort **Worst Possible Pain**

P4. Which of the following statements best describe your experience regarding <u>physical activities</u> and the pain or discomfort in relation to your kidney problem?

- I **do not experience any pain** or discomfort during physical activities ☐ 1
- I experience pain or discomfort only when I perform **vigorous activities** (e.g. strenuous sports, lifting heavy objects) ☐ 2
- I experience pain when I perform **activities of moderate severity** but not with basic activities (e.g. walking few hundred yards, driving a car) ☐ 3
- I experience pain even when I perform **basic activities** of daily living (e.g. walking indoors, dressing) ☐ 4
- I experience pain while also **being at rest** ☐ 5

P5. Does the pain or discomfort, in relation to your kidney problem, interrupt your sleep?

- Never ☐ 1
- Occasionally ☐ 2
- Sometimes ☐ 3
- Most of the time ☐ 4
- All of the time ☐ 5

P6. Do you experience pain or discomfort, in relation to your kidney problem, while passing urine?

- Never ☐ 1
- Occasionally ☐ 2
- Sometimes ☐ 3
- Most of the time ☐ 4
- All of the time ☐ 5

P7. Do you experience pain or discomfort in the <u>kidney area</u>, while passing urine?

- No ☐ 1
- Yes ☐ 2

P8. How frequently have you required painkillers to control the pain or discomfort associated with the kidney problem?

- Never ☐ 1
- Occasionally ☐ 2
- Sometimes ☐ 3
- Most of the time ☐ 4
- All of the time ☐ 5

P9. Overall, how much does the pain or discomfort, in relation to your kidney problem, (as distinct from other symptoms) interfere with your life?

- Not at all ☐ 1
- A little bit ☐ 2
- Moderately ☐ 3
- Quite a bit ☐ 4
- Extremely ☐ 5

Please go to next section --

GENERAL HEALTH:

Following removal of the stent:

G1. Have you had difficulty in performing light physical activities (e.g. walking short distances, driving a car)?

- Usually with no difficulty ☐ 1
- Usually with some difficulty ☐ 2
- Usually with much difficulty ☐ 3
- Usually did not do because of the stent ☐ 4
- Usually did not do for other reasons ☐ 0

G2. Have you had difficulty in performing heavy physical activities (e.g. strenuous sports, lifting heavy objects)?

- Usually with no difficulty ☐ 1
- Usually with some difficulty ☐ 2
- Usually with much difficulty ☐ 3
- Usually did not do because of the stent ☐ 4
- Usually did not do for other reasons ☐ 0

G3. Have you felt tired and worn out?

- Never ☐ 1
- Occasionally (less than one third of the time) ☐ 2
- Sometimes (between one and two thirds of the time) ☐ 3
- Most of the time (more than two thirds of the time) ☐ 4
- All of the time ☐ 5

G4. Have you felt calm and peaceful?

- All of the time ☐ 1
- Most of the time ☐ 2
- Sometimes ☐ 3
- Occasionally ☐ 4
- Never ☐ 5

G5. Have you enjoyed your social life (going out, meeting friends and so on)?

- All of the time ☐ 1
- Most of the time ☐ 2
- Sometimes ☐ 3
- Occasionally ☐ 4
- Never ☐ 5

G6. Have you needed extra help from your family members or friends?

- Never ☐ 1
- Occasionally ☐ 2
- Sometimes ☐ 3
- Most of the time ☐ 4
- All of the time ☐ 5

Please go to next section ---

WORK PERFORMANCE:

W1. Regarding your employment status, are you

In full time employment ☐ 1
In part time employment ☐ 2
Retired on health ground ☐ 3
Student ☐ 4
Unemployed, looking for work ☐ 5
Retired for other reason (including age) ☐ 6
Not working for other reason (please specify) ☐ 7 ________________________________

W2. Following removal of the stent, how many days did the symptoms associated with the kidney problem keep you in bed all or most of the day or result in the loss of a full day's work?

☐☐ Day(s)

W3. Following removal of the stent, for how many <u>half days</u> did you cut down your routine activities (including work) because of the symptoms associated with the kidney problem?

☐☐ Half Day(s)

Please answer the questions below (W4 –W7) only if you are in active paid work.

(Otherwise ignore questions W4 – W7).

W4. a) Job title or description of your role: ________________________________

b) Are you an: Employee ☐ 1 Employer ☐ 2 Self employed ☐ 3

<u>Please answer following questions if you have worked after removal of the stent,</u>

W5. Have you worked for short periods of time or taken frequent rests because of the symptoms associated with the kidney problem?

Never ☐ 1
Occasionally ☐ 2
Sometimes ☐ 3
Most of the time ☐ 4
All of the time ☐ 5

W6. Have you worked at your usual job, but with some changes because of the symptoms associated with the kidney problem?

Never ☐ 1
Occasionally ☐ 2
Sometimes ☐ 3
Most of the time ☐ 4
All of the time ☐ 5

W7. Have you worked your regular number of hours?

Never ☐ 5
Occasionally ☐ 4
Sometimes ☐ 3
Most of the time ☐ 2
All of the time ☐ 1

Please go to next section --

SEXUAL MATTERS:

Please tick one box for each question by thinking about **your experience following removal of the stent**.

S1. Currently, do you have an active sex life?

No ☐ 1, Please **answer question S2 and go to the last question GQ.**

Yes ☐ 2, Please go to question S3 (**Ignore question S2**).

S2. (i) If NO sex life, how long ago did this stop?

After insertion of the stent ☐ 1

Before insertion of the stent ☐ 0

(ii)AND, why did this stop?

Because of the kidney problem ☐ 10

Did not attempt any sexual activity ☐ 0

Some other reason – not to do with the kidney problem ☐ 0

(Ignore questions S3 – S4)

Please answer questions S3 and S4, only if you have answered 'yes' to question S1.

Please think about your experience following removal of the stent.

S3. Do you have pain when you have sexual intercourse?

Not at all ☐ 1

Mild ☐ 2

Moderate ☐ 3

Severe ☐ 4

Extreme ☐ 5

S4. How satisfied are you with your sex life?

Very satisfied ☐ 1

Satisfied ☐ 2

Not sure ☐ 3

Dissatisfied ☐ 4

Very dissatisfied ☐ 5

GQ In the future, if you were advised to have another stent inserted, how would you feel about it?

Delighted ☐ 1

Pleased ☐ 2

Mostly satisfied ☐ 3

Mixed feelings (about equally satisfied and dissatisfied) ☐ 4

Mostly dissatisfied ☐ 5

Unhappy ☐ 6

Terrible ☐ 7

AQ. If there are any comments you would like to make about the questionnaire or any of your symptoms, please use the space below.

THANK YOU VERY MUCH FOR YOUR HELP

(Please complete the enclosed questionnaires)
