# Supplementary material for: Striking a balance: outcomes of short-term Mono-J placement following ureterorenoscopy
Source: Urolithiasis. 2021 Apr 13;49(6):567–73. doi: 10.1007/s00240-021-01264-4 (PMC8560726; doi:10.1007/s00240-021-01264-4)
Supplement: Supplementary file 2 — Supplementary file2 (PDF 101 KB) [file 240_2021_1264_MOESM2_ESM.pdf]

# URETERIC
# STENT SYMPTOMS QUESTIONNAIRE

## Questionnaire 1 ( *Stent in situ* )

**We are interested to know about various aspects of <u>your health, following insertion of the stent</u> and the effect the stent has had on your health.**

**Please complete the following questionnaire, which has different sections. Please answer all questions in each section.**

**(We would be grateful if you could complete and post the questionnaire within seven days)**

**Please complete:**

**Today's Date:** ☐☐ / ☐☐ / ☐☐

**Date of Birth:** ☐☐ / ☐☐ / ☐☐

***Please return to:***

**Post Code:** ☐☐☐☐ ☐☐☐
**Hospital Number :** ☐☐☐☐☐☐☐☐
**(for office use)**

You will see that some questions ask if you have a symptom occasionally, sometimes or most of the time.

| | | |
|---|---|---|
| **Occasionally** | **=** | **less than one third of the time** |
| **Sometimes** | **=** | **between one and two thirds of the time** |
| **Most of the time** | **=** | **more than two thirds of the time** |

## URINARY SYMPTOMS

Please answer the questions thinking about the urinary symptoms you have experienced following insertion of the stent.

Please put a tick in one box for each question ☑

**Please think about your experience since insertion of the stent.**

**U1. During the day, how often do you pass urine, on average?**

- Less than hourly ☐ 5
- Hourly ☐ 4
- Every 2 hourly ☐ 3
- Every 3 hourly ☐ 2
- Every 4 hours or more ☐ 1

**U2. During the night, how many times do you have to get up to pass urine, on average?**

- None ☐ 1
- 1 ☐ 2
- 2 ☐ 3
- 3 ☐ 4
- 4 or more ☐ 5

**U3. Do you have to rush to the toilet to urinate?**

- Never ☐ 1
- Occasionally ☐ 2 (less than one third of the time)
- Sometimes ☐ 3 (between one and two thirds of the time)
- Most of the time ☐ 4 (more than two thirds for the time)
- All of the time ☐ 5

**U4. Does urine leak before you can get to the toilet?**

- Never ☐ 1
- Occasionally ☐ 2
- Sometimes ☐ 3
- Most of the time ☐ 4
- All of the time ☐ 5

**U5. Do you leak urine without feeling the need to go to the toilet?**

- Never ☐ 1
- Occasionally ☐ 2
- Sometimes ☐ 3
- Most of the time ☐ 4
- All of the time ☐ 5

**U6. how often do you feel that your bladder has not emptied properly after you have passed urine?**

- Never ☐ 1
- Occasionally (less than one third of the time) ☐ 2
- Sometimes (between one and two thirds of the time) ☐ 3
- Most of the time (more than two thirds for the time) ☐ 4
- All of the time ☐ 5

**U7. Do you have a burning feeling when you pass urine?**

- Never ☐ 1
- Occasionally ☐ 2
- Sometimes ☐ 3
- Most of the time ☐ 4
- All of the time ☐ 5

**U8. How often do you blood in your urine?**

- Never ☐ 1
- Occasionally ☐ 2
- Sometimes ☐ 3
- Most of the time ☐ 4
- All of the time ☐ 5

**U9. How much blood do you see in your urine?**

- Do not see any blood ☐ 1
- Urine is slightly blood stained ☐ 2
- Urine is heavily blood stained ☐ 3
- Urine is heavily blood stained and has clot(s) ☐ 4

**U10. Overall, how much of a problem are your urinary symptoms to you?**

- Not at all ☐ 1
- A little bit ☐ 2
- Moderate ☐ 3
- Quite a bit ☐ 4
- Extreme ☐ 5

**U11. If you were to spend the rest of your life with the urinary symptoms, if any, associated with the stent just the way they are, how would you feel about it?**

- Delighted ☐ 1
- Pleased ☐ 2
- Mostly satisfied ☐ 3
- Mixed feelings (about equally satisfied and dissatisfied ☐ 4
- Mostly dissatisfied ☐ 5
- Unhappy ☐ 6
- Terrible ☐ 7

Please go to next section --

## BODY PAIN (for women):

This section asks about the **body pain or discomfort, which you associate with the stent.**

Please think about your experience **following insertion of the stent.**

**P1. Do you experience body pain or discomfort in association with the stent?**

**YES** ☐1, **please go to question P2**

**NO** ☐2, **please go to next section on General Health** (Ignore questions P2 to P9)

**P2.** Think of the drawings below as the drawings of your body. Please **mark (X) or shade the site(s) where you experience pain or discomfort in association with the stent typically (e.g. during the day to day activities, whenever you pass urine)**

If you get pain at more than one site, please use a separate mark for each site.

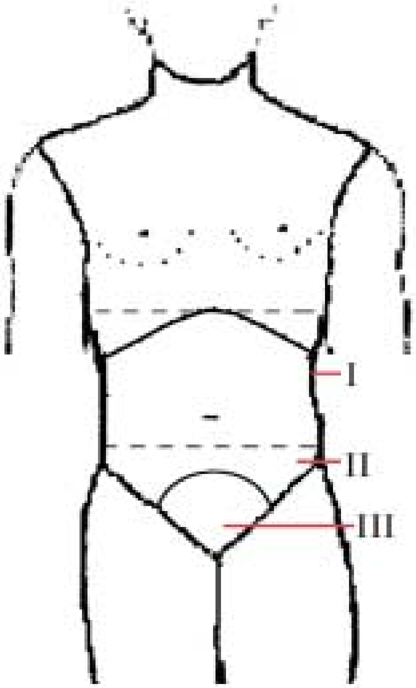


Front View

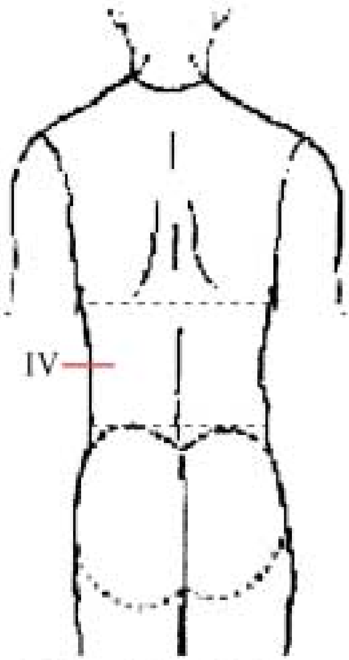


Back View

**The numbers I - IV represent following areas for the right and left sides**

**I** – Kidney front/side area

**II** - Groin area

**III** – Bladder area

**IV**– Kidney back (loin) area

**Please use O** for any other marked area and write the name of the site

**P3.** Please place a mark (X) to a point on the line below that indicates your pain or discomfort in association with the stent. **Please put a separate mark for each site** if the pain or discomfort is different in severity and write the corresponding number of each site used in the drawing above**.**

0 1 2 3 4 5 6 7 8 9 10

**No Pain or discomfort** **Worst Possible Pain**

## BODY PAIN (for men):

This section asks about the **body pain or discomfort, which you associate with the stent.**

Please think about your experience **following insertion of the stent.**

**P1. Do you experience body pain or discomfort in association with the stent?**

**YES** ☐1, **please go to question P2**

**NO** ☐2, **please go to next section on General Health** (Ignore questions P2 to P9)

**P2.** Thinking the drawings below as the drawings of your body, **mark (X) or shade the site(s) where you experience pain or discomfort in association with the stent typically (e.g. during the day to day activities, whenever you pass urine)**

If you get pain at more than one site, please use a separate mark for each site.

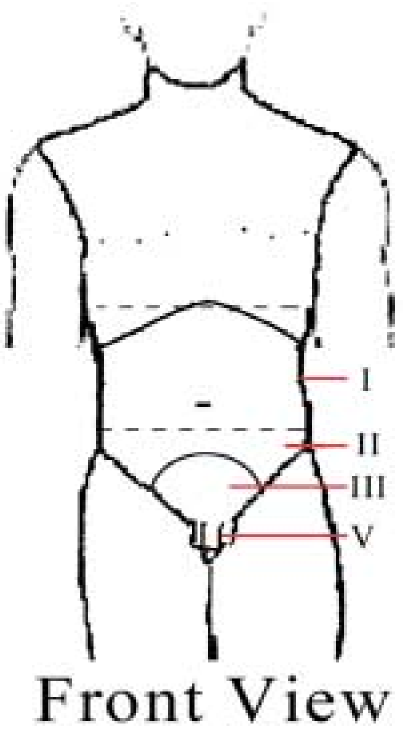


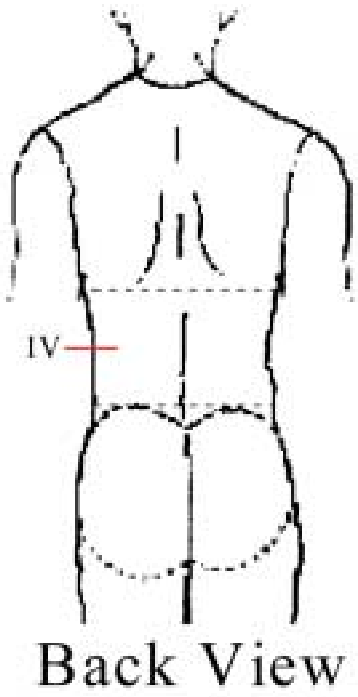


**The numbers I - V represent following areas for the right and left side.**

**I** – Kidney front/side area
**IV**– Kidney back (loin) area
**II** - Groin area
**V** – Penis
**III** – Bladder area
Please use **O** for any other marked site and name that site.

**P3.** Please place a mark (X) to a point on the line below that indicates your pain or discomfort in association with the stent. **Please put a separate mark for each site** if the pain or discomfort is different in severity and write the corresponding number of each site used in the drawing above.

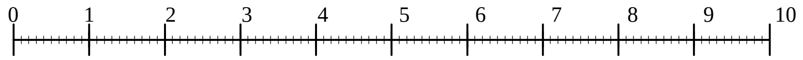


**No Pain or discomfort** **Worst Possible Pain**

**P4. Which of the following statements best describe your experience regarding physical activities and the pain or discomfort in association with the stent?**

I **do not experience any pain** or discomfort during physical activities ☐ 1

I experience pain or discomfort only when I perform **vigorous activities** ☐ 2
(e.g. strenuous sports, lifting heavy objects)

I experience pain when I perform **activities of moderate severity** but not with basic activities ☐ 3
(e.g. walking few hundred yards, driving a car)

I experience pain even when I perform **basic activities** of daily living ☐ 4
(e.g. walking indoors, dressing)

I experience pain while also **being at rest** ☐ 5

**P5. Does the pain or discomfort, in association with the stent, interrupt your sleep?**

Never ☐ 1
Occasionally ☐ 2
Sometimes ☐ 3
Most of the time ☐ 4
All of the time ☐ 5

**P6. Do you experience pain or discomfort, in association with the stent, while passing urine?**

Never ☐ 1
Occasionally ☐ 2
Sometimes ☐ 3
Most of the time ☐ 4
All of the time ☐ 5

**P7. Do you experience pain or discomfort in the kidney area, while passing urine?**

No ☐ 1
Yes ☐ 2

**P8. How frequently have you required painkillers to control the pain or discomfort associated with the stent?**

Never ☐ 1
Occasionally ☐ 2
Sometimes ☐ 3
Most of the time ☐ 4
All of the time ☐ 5

**P9. Overall, how much does the pain or discomfort, in association with the stent, (as distinct from other symptoms) interfere with your life?**

Never ☐ 1
Occasionally ☐ 2
Sometimes ☐ 3
Most of the time ☐ 4
All of the time ☐ 5

Please go to next section --

## GENERAL HEALTH:

**Following insertion of the stent:**

**G1. Have you had difficulty in performing light physical activities (e.g. walking short distances, driving a car)?**

- Usually with no difficulty ☐ 1
- Usually with some difficulty ☐ 2
- Usually with much difficulty ☐ 3
- Usually did not do because of the stent ☐ 4
- All of the time ☐ 0

**G2. Have you had difficulty in performing heavy physical activities (e.g. strenuous sports, lifting heavy objects)?**

- Usually with no difficulty ☐ 1
- Usually with some difficulty ☐ 2
- Usually with much difficulty ☐ 3
- Usually did not do because of the stent ☐ 4
- All of the time ☐ 0

**G3. Have you felt tired and worn out?**

- Never ☐ 1
- Occasionally (less than one third of the time) ☐ 2
- Sometimes (between one and two thirds of the time) ☐ 3
- Most of the time (more than two thirds of the time) ☐ 4
- All of the time ☐ 5

**G4. Have you felt calm and peaceful?**

- All of the time ☐ 1
- Most of the time (less than one third of the time) ☐ 2
- Sometimes (between one and two thirds of the time) ☐ 3
- Occasionally (more than two thirds of the time) ☐ 4
- Never ☐ 5

**G5. Have you enjoyed your social life ( going out, meeting friends and so on )?**

- All of the time ☐ 1
- Most of the time ☐ 2
- Sometimes ☐ 3
- Occasionally ☐ 4
- Never ☐ 5

**G6. Have you needed extra help from your family members or friends?**

- Never ☐ 1
- Occasionally ☐ 2
- Sometimes ☐ 3
- Most of the time ☐ 4
- All of the time ☐ 5

Please go to next section –

## WORK PERFORMANCE:

**W1. Regarding your employment status, are you**

In full time employment ☐ 1 — Student ☐ 4

In part time employment ☐ 2 — Unemployed, looking for work ☐ 5

Retired on health ground ☐ 3 — Retired for other reason (including age) ☐ 6

Not working for other reason (please specify) ☐ 7 ______________________________

**W2. Following insertion of the stent, how many days did the symptoms associated with the stent keep you in bed all or most of the day?**

☐☐ Day(s)

**W3. Following insertion of the stent, for how many half days or more did you cut down your routine activities because of the symptoms associated with the stent?**

☐☐ Half Day(s)

**Please answer the questions below (W4 –W7 ) only if you are in active paid work.**

**(Otherwise ignore questions W4 – W7).**

**W4.** a) Job title or description of your role: ______________________________

b) Are you an: Employee ☐ 1 Employer ☐ 2 Self employed ☐ 3

**<u>Please answer following questions if you have worked after insertion of the stent,</u>**

**W5. Have you worked for short periods of time or taken frequent rests because of the symptoms associated with the stent?**

Never ☐ 1 — Most of the time ☐ 4

Occasionally ☐ 2 — All of the time ☐ 5

Sometimes ☐ 3

**W6. Have you worked at your usual job, but with some changes because of the symptoms associated with the stent?**

Never ☐ 1 — Most of the time ☐ 4

Occasionally ☐ 2 — All of the time ☐ 5

Sometimes ☐ 3

**W7. Have you worked your regular number of hours?**

Never ☐ 1 — Most of the time ☐ 4

Occasionally ☐ 2 — All of the time ☐ 5

Sometimes ☐ 3

**Please go to next section –**

## SEXUAL MATTERS:

Please tick one box for each question by thinking about **your experience following insertion of the stent**.

**S1. Currently, do you have an active sex life?**

**No** ☐ 1, Please answer question S2 and go to next section (Ignore questions S3 and S4).

**Yes** ☐ 2, Please go to question S3 (Ignore question S2).

**S2. (i) If NO sex life, how long ago did this stop?**

| | | |
|---|---|---|
| After insertion of the stent | ☐ | 1 |
| Before insertion of the stent | ☐ | 0 |

**(ii)AND, why did this stop?**

| | | |
|---|---|---|
| Because of the problems associated with the stent | ☐ | 10 |
| Did not attempt any sexual activity | ☐ | 0 |
| Some other reason – not to do with the symptoms of the stent | ☐ | 0 |

**( Ignore questions S3 – S4 )**

***Please answer questions S3 and S4, only if you have answered 'yes' to question S1.***

**Please think about your experience following insertion of the stent.**

**S3. Do you have pain when you have sexual intercourse?**

| | | | | | |
|---|---|---|---|---|---|
| Not at all | ☐ | 1 | Severe | ☐ | 4 |
| Mild | ☐ | 2 | Extreme | ☐ | 5 |
| Moderate | ☐ | 3 | | | |

**S4. How satisfied are you with your sex life?**

| | | | | | |
|---|---|---|---|---|---|
| Very satisfied | ☐ | 1 | Dissatisfied | ☐ | 4 |
| Satisfied | ☐ | 2 | Very dissatisfied | ☐ | 5 |
| Not sure | ☐ | 3 | | | |

Please go to next section ---

## ADDITIONAL PROBLEMS:

**The following questions ask about your experience following insertion of the stent. Please indicate your experience by ticking the appropriate box.**

**A1. How many times have you felt you may be suffering from a urinary tract infection (e.g. running temperature, feeling unwell and pain while passing urine)?**

- Never ☐ 1
- Occasionally ☐ 2
- Sometimes ☐ 3
- Most of the time ☐ 4
- All of the time ☐ 5

**A2. Have you needed to take antibiotics as a result of insertion of the stent? ( Please ignore the course of antibiotics, which may have been given at the time of insertion of the stent. )**

- Not at all ☐ 1
- One Course ☐ 2
- Two Courses ☐ 3
- Three or more Courses ☐ 4

**A3. Have you needed to seek help of a health professional (such as GP, nurse) due to any problem associated with the stent?**

- Never ☐ 1
- Once ☐ 2
- Twice ☐ 3
- Three or more times ☐ 4

**A4. Have you needed to visit the hospital due to any problem associated with the stent?**

- Never ☐ 1
- Once ☐ 2
- Twice ☐ 3
- Three or more times ☐ 4

**GQ In the future, if you were advised to have another stent inserted, how would you feel about it?**

- Delighted ☐ 1
- Pleased ☐ 2
- Mostly satisfied ☐ 3
- Mixed feelings (about equally satisfied and dissatisfied) ☐ 4
- Mostly dissatisfied ☐ 5
- Unhappy ☐ 6
- Terrible ☐ 7

**AQ. If there are any comments you would like to make about the questionnaire or any of your symptoms, please use the space below**.

THANK YOU VERY MUCH FOR YOUR HELP

All information will remain confidential
